# The analysis of hippocampus neuronal density (CA1 and CA3) after *Ocimum sanctum* ethanolic extract treatment on the young adulthood and middle age rat model

**DOI:** 10.14202/vetworld.2018.135-140

**Published:** 2018-02-08

**Authors:** Dwi Liliek Kusindarta, Hevi Wihadmadyatami, Aris Haryanto

**Affiliations:** 1 Department of Anatomy, Faculty of Veterinary Medicine, Universitas Gadjah Mada, Yogyakarta, Indonesia; 2 Department of Biochemistry, Faculty of Veterinary Medicine, Universitas Gadjah Mada, Yogyakarta, Indonesia

**Keywords:** acetylcholine, CA1, CA3, *Ocimum sanctum*

## Abstract

**Aim:**

This study aimed to assess the changes in neuronal density in CA1 and CA3 regions in the hippocampus of young adulthood and middle age rat model after feeding by *Ocimum sanctum* ethanolic extract.

**Materials and Methods:**

In this research, 30 male Wistar rats consist of young to middle-aged rats were divided into three groups (3, 6, and 9 months old) and treated with a different dosage of *O. sanctum* ethanolic extract (0, 50, and 100 mg/kg b.w.) for 45 days. Furthermore, cresyl violet staining was performed to analyze hippocampus formation mainly in CA1 and CA3 area. The concentrations of acetylcholine (Ach) in brain tissues were analyzed by enzyme-linked immunosorbent assay.

**Results:**

In our *in vivo* models using rat model, we found that the administration of *O. sanctum* ethanolic extract with a dosage of 100 mg/kg b.w. for 45 days induced the density of pyramidal cells significantly in CA1 and CA3 of the hippocampus. These results were supported by an increase of Ach concentrations on the brain tissue.

**Conclusions:**

The administration of *O. sanctum* ethanolic extract may promote the density of the pyramidal cells in the CA1 and CA3 mediated by the up-regulated concentration of Ach.

## Introduction

In recent year deterioration in cognitive, learning, and memory become one of the significant problems in human life. Hippocampus is a pivotal part of the brain’s limbic system which serves a critical role in memory, learning process and regulating the emotions. CA1 and CA3 of the hippocampus is an area which has responsibility for matching sensory input with context-dependent retrieval for memory thus may guide behavior in different tasks. The dysfunction of CA 1 and CA3 area cause of aging or pathologic condition such as stroke vascular, Parkinson and Alzheimer disease, could promote neurodegeneration disease which leads dementia as a clinical manifestation. To address the behavior differences between the age cohorts, the large number of studies was investigated between the young and aged animals in recent years [1-3]. These studies suggested significant differences were observed in various behaviors between age cohorts, indicating that aging is contributing to behavioral impairment [4]. However, only a few researches describe the detailed mechanism on the central nervous system regarding the deterioration of cognitive ability. Moreover, the effect of age on behavior during the early stages of life in the absence of disease needs to be understood.

*Ocimum sanctum* belongs to the family Lamiaceae, is one of the potential herbs used for medication. Almost parts of *O. sanctum* now extensively accepted and give several advantages as an anti-inflammatory [5], antiallergic [6], antioxidant [7,8], radioprotective [9], and also as anticarcinogenic [10]. Moreover, recently we also reported that *O. sanctum* maintained the expression of choline acetyltransferase in human brain microvascular endothelial cells in aging condition [11].

This present study was designed to analyze the ability of an ethanolic extract derived from the leaf of *O. sanctum* to stimulate the activity of acetylcholine (Ach) as a neurotransmitter in neuronal cells. We postulated that increasing concentration of Ach might promote increasing the density of pyramidal cells in CA1 and CA3 of the hippocampus in young middle-age individuals.

## Materials and Methods

### Ethical approval

The use of all preclinical research material was approved by the Ethics Committee of Universitas Gadjah Mada, Yogyakarta, Indonesia, number: 00060/04/LPPT/IX/2016.

### Crude and ethanolic extract of O. sanctum

*O. sanctum* leaves were prepared from Center for Research and Development of Medicinal Plants and Traditional Medicines, Ministry of Health in Tawangmangu, Central Java, Indonesia. Crude extracts and ethanolic extracts of *O. sanctum* were prepared as previously described [11].

### Animal model

We used in total 30 male, white rats (Wistar rats), in a variation of ages 3 months old (represents 9 years old in human), 6 months (represents 18 years old in human), and 9 months (represents 25 years old in human) as previously described [12,13]. The rats were derived from Biofarma, Bandung, West Java, Indonesia, and had 250-300 g average of weights. They kept under standard conditions with free access to food and water 12/12 h daylight/dark. The rats were divided into three groups (n=3) based on the dosage of *O. sanctum* (0 mg/kg, 50 mg/kg, and 100 mg/kg b.w.). The ethanolic extract of *O. sanctum* was given daily for 45 days.

### Hippocampal brain section

Animals were anesthetized using a combination of 10% ketamine hydrochloride (Ketamil ilium, TroyLab, Glenningen, New South Wales, and Australia) and xylazine 2% (Xyla, Interchemie, Venray, and the Netherland) intramuscularly. Perfusion performed using pre-rinse fluid. Rat body placed dorsal, fixed, and slightly moistened in the hair. The thoracic cavity is opened with scissors until the color is appeared for perfusion and followed by introducing pre-rinse liquid (0.9% NaCl with 0.1 g of NaCl at 100 ml of distilled water, 1 ml EDTA) through the sinister ventricle, the auricle at the Dexter atrium is cut out so that the liquid pre-rinse can come out. Fixation using 4% formalin phosphate buffered saline (PBS) solution intracardially after the pre-rinse fluid became clear. Brain removal is done by slashing the head scalp until the cranium appears. The scissors are inserted in the foramen magnum and shear the lateral part toward the acusticus porous, opening the os occipitalis and visible the cerebellum. The temporal os is cut from the porous acoustic Dexter and the sinister toward the nose. The parietal os opened slowly. The brain is then separated from its cranium by cutting off the cranial nerve and the olfactory bulb tip.

### Tissue processing

The collected brain was fixed by formalin PBS 4% for 45 min. The brain then washed using PBS (pH 7.4) for 10 min and followed with 30% sucrose solution for 24 h at 4°C. Trimming were proceed by cutting the hippocampus section with a size of 1×1 cm. The hippocampus is inserted in FSC 22^®^ (Leica, Wetzlar, Germany) and put on an aluminum foil chamber and allowed to stand for 1-2 h. The hippocampus block was frozen in a freezer with a temperature of −80°C. Frozen hippocampal block then cuts using Cryostat (Leica, Wetzlar, Germany) at −25°C with a thickness of 20 μm and attached to gelatin-coated slides. The slides were saved at −40°C until it used for staining.

### Cresyl violet staining and cell counting

The cresyl violet (Sigma, Steinheim, Germany) solution is filtered with filter paper and put in an incubator at 37°C. On the next day, the slides are removed from the freezer and then settled at room temperature for 1-2 h until the temperature fits the room temperature. The slides are inserted into an incubator at 37°C for 5 min and then washed with PBS (Thermo Fischer, Rockford, IL, USA) 5 min. The slides are arranged on a rack and stained with cresyl violet in an incubator for 30 min. The slides rinsed with Aquades 1 min, then dehydrated using Ethanol 70%, 80%, and 90%, absolute ethanol I and II, followed by xylene I, II, and III. Slide then mounting with balsam Canada and covering it with a cover glass. The formation of the hippocampus was observed using a light microscope (Nikon, Tokyo, Japan) at the magnification of 40 fold. Data analysis was done by microscopic readings software Optilab Image Viewer (Optilab, Yogyakarta, Indonesia). The amount of the cell in the hippocampus area (mm^2^) calculated by Optilab Image Raster software (Optilab, Yogyakarta, Indonesia).

### Ach enzyme-linked immunosorbent assay (ELISA)

The concentration of Ach on rat brain was determined using ELISA kit (Elabscience, Houston, Texas, USA). Brain tissues were minced to small pieces and rinsed in ice-cold PBS (pH=7.4). Tissue pieces weighed and homogenized in PBS. Sonication was done for 10 min, and the homogenates were then centrifuged for 5 min at 5000×*g* to get the supernatant. An amount of 50 µl standard, sample and control were put into a precoated well plate and followed by the addition of 50 µl biotin detection antibody working solution in each well. The well plates were incubated for 45 min in 37°C. All solutions were aspired, followed by 3 times washing procedure using wash buffer. After the final wash remaining wash buffer was removed by decanting. Then, 100 µl HRP conjugate working solutions were added and incubated for 30 min at 37°C. After 5 times washing procedure, 90 µl of substrate reagent was added to each well and incubated for 15 min at 37°C in dark chamber. After reaction termination using 50 µl stop solution optical density (OD) value was determined using microplate reader at 450 nm.

### Statistics

Statistical analysis was made using an unpaired, 2-tailed Student’s t-test as appropriate. p<0.05 was assumed to represent statistical significance. Statistic was performed by using GraphPad Prism 6 (La Jolla, CA, USA).

## Results

### The pyramidal cells on CA1 and CA3 decreasing gradually following increasing the ages on rat model

**Figure-1:**
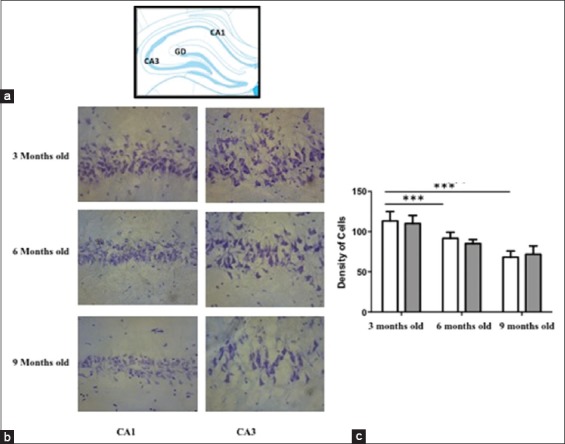
(a) The hippocampus schematic picture (modification from Paxinos). (b) The estimation of pyramidal cells density in the CA1 and CA3 of the untreated rat model. The cells on the brain tissue were stain using cresyl violet. A significantly decreasing of pyramidal cells was found on 6 months old and 9 months old rat model both on CA1 and CA3 in comparison to the young adulthood rat model (p<0.05). (c) The diagram visualized the density of pyramidal cells. White column=CA1 area; Gray column=CA3 area.

Following the tissue processing, cells on the brain tissue were stain with cresyl violet. This staining describes that there is a significantly decreasing amount of pyramidal cells on the pyramidal cell layer (PCL) of CA1 and CA3 area. Following the tissue processing, cells on the brain tissue were stain with cresyl violet. This staining describes that there is a significantly decreasing amount of pyramidal cells on the pyramidal cell layer (PCL) of CA1 and CA3 area, following the increasing of the ages on the rat model (3 months old, 6 months old and 9 months old) (Figure-1a, b, and c).

### Ethanolic extract of O. sanctum capable to maintain and promote increasing of pyramidal cell on the young adulthood and middle age rat model

**Figure-2:**
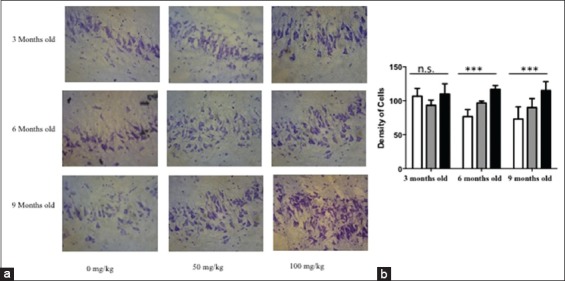
The estimation of pyramidal cells density in the CA1 of the young adulthood and middle-aged treated rat model. Rat models were treated by ethanolic extract of *O. sanctum*. Followed by tissue processing cresyl violet staining was performed to described the pyramidal cells on the pyramidal cell layer on CA1 area. A significantly increasing density of pyramidal cells was found in the CA 1 area on the middle age rat model (p<0.05). (a) The photomicrograph of CA1 area. (b) The diagram visualized the density of pyramidal cells. White column=0 mg/kg b.w.; Gray column=50 mg/kg b.w.; Black column=100 mg/kg b.w.; magnification 40×, scale bar 40 µm, n.s.=Non-significant.

**Figure-3:**
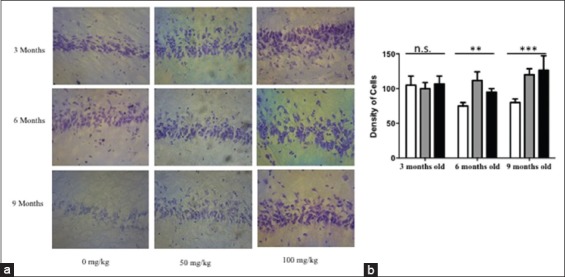
The amount of pyramidal cells density in the CA3 of the young adulthood and middle-aged treated rat model. Rat models were treated by ethanolic extract of *Ocimum sanctum*. Cresyl violet was performed to describe the density of pyramidal cells on the pyramidal cell layer on CA1 area. A significantly increasing density of pyramidal cells was found in the CA3 area on the middle age rat model (p<0.05). (a) The photomicrograph of CA3 area. (b) The diagram visualized the density of pyramidal cells. White column=0 mg/kg b.w.; Gray column=50 mg/kg b.w.; Black column=100 mg/kg b.w.; magnification 40×, scale bar 40 µm, n.s=non-significant.

After treatment of an ethanolic extract derived from the leafs of *O. sanctum* hippocampus were stained using cresyl violet. On the untreated middle age rat model (6 months old and 9 months old) we can describe that there is decreasing the amount of pyramidal cells in the CA1 and CA 3 (Figures-2 and 3), in contrast on the treated middle age rat model, observed that ethanolic extract *O. sanctum* capable to stimulate increasing of pyramidal cells (Figures-2 and 3). In addition, on the young adulthood rat model (3 months old) ethanolic extract *O. sanctum* could maintain the amount of the pyramidal cells (Figures-2 and 3).

### Upregulated concentration of Ach was found on the brain tissue of middle age rat model after treatment with ethanolic extract of O. sanctum

**Figure-4:**
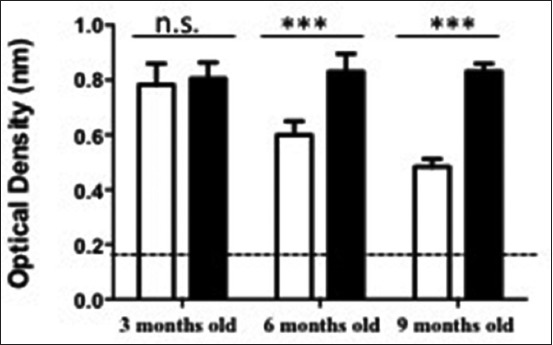
Acetylcholine (Ach) activity on the brain tissue with and without treatment of ethanolic extract *Ocimum sanctum* Linn. Indirect Ach enzyme-linked immunosorbent assay from brain tissue was performed to analysis the Ach concentration. Statistical analysis was performed by two-tailed Student’s t-test. All experiment were performed in duplicate (n=3. White column=Untreated rat model; Black column=Treated rat model; n.s.=Non-significant).

To analyze the concentration of Ach on the young adulthood and middle age rat model after treatment, sandwich ELISA was performed. The ELISA absorbance results could be described that there is a significantly upregulated Ach concentration on the middle age rat model (6 months old and 9 months old) after treatment (OD; 0.830 and 0.833) as same as young age rat model (OD; 0.783). In the other hand, Ach concentration on the young adulthood rat model (3 months old) is precisely maintain in the same concentration in the presence of an ethanolic extract of *O. sanctum* (OD; 0.783 vs. 0.805) (Figure-4).

## Discussion

For decades, it has been mentioned that widespread neuron death in the neocortex and hippocampus is an ineluctable concomitant of brain ageing cause of diseases. However, recent studies suggest that neuron death also occurs in functional aging and its seems in related to an impairment of neocortical and hippocampal functions during aging processes [14]. Data from WHO and Alzheimer report show increasing people suffering from dementia along with aging. Recently, dementia not only suffered by elderly or old people but also individual in the early stage of life (young adulthood to middle age). This phenomenon may occur because of the lifestyle condition of the modern people. Dementia is one of clinical manifestation from cognitive and memory impairment. Medication which promotes for curing dementia usually only administrated after the neurodegeneration diseases occur, thus it did not give any satisfying results. That is why the new finding of drugs to treat dementia is crucial not only for medication but also for prevention of disease. *O. sanctum* is one of herb which easies to find especially in the tropical and subtropical country. Some data already mentioned and describe *O. sanctum* have much beneficent as antioxidant, anticancer, and anti-inflammation, and some research also reported an ethanolic extract from *O. sanctum* probably might act as neuroprotection [6,15]. Despite its potential role in different diseases, until today restricted data are available regarding the preventive and curing effects of *O. sanctum* in cognitive impairment mainly with dementia as a clinical sign.

In our study, we already found that there is a decreasing amount of pyramidal cells on CA 1 and CA3 gradually from 6 months old rats (24 weeks old rat) to 9 months old rats (36 weeks old rat). When we administrated 100 mg/kg b.w. of ethanolic extract *O. sanctum* for 45 days showed a significant improvement in the pyramidal cell density in the CA1 and CA3 area of 9 months old rats, as same as on the young adult rats (3 months old) (Figures-1 and 2). Of note, in here 9 months old rats are equal to 25 years old human (adult) whereas 3 months old as same as like 9 years old human [12,13]. Decreasing amount of pyramidal cells in CA1 and CA3 on the rat without treatment are in line with another evidence, which is mention that numerical density of pyramidal cells on the CA1 and CA4 on the 5-21 months old rats are less than the amount of the cells on the 1-4 months old rats [16-18]. The other research observes that the spatial learning and memory functions were declined from 8 to 12 months of age in C57BL/6J mice [4]. From the functional description, the CA1 region is an area which plays an essential role to participate in new memory formation, and memory strengthens [19,20]. Whereas, CA3 represents the primary component of CA1 activity through Schaffer collaterals pathway which may guarantee the sustainability signal from entorhinal cortex to reach CA1 region. The connectivity between CA1 and CA3 may enhance the long-term potentiation related to learning memory ability in the healthy individual [21,22]. In addition, CA1 neuron number has been found related to the severity of dementia [23]. Thus, the sustainability pyramidal cells in CA1 and CA3 become important thinks. The intriguing question is how the ethanolic extract of *O. sanctum* may maintain and or promote increasing the density of pyramidal cell in CA1 and CA3?

To answer the question, we propose an experiment using ELISA to analyze the concentration of neurotransmitter Ach on the brain tissues. In the present study, we found that in the treatment of 100 mg/kg b.w. ethanolic extract *O. sanctum*, the middle-aged rats (6 and 9 months old) showed a higher concentration of Ach in comparison to the young adult age group (3 months old) (Figure-3). The increasing Ach concentration also found when we give 50 mg/kg b.w. ethanolic extract to the rat. However, there is no significant difference between treated and untreated rat (Figure-3). Already known Ach is one of classical neurotransmitter which runs as a regulator of neuronal cells neurogenesis and differentiation [24-27]. Indeed, some investigation also reported that the cholinergic system played an important role in learning and memory [28,29]. More importantly, Ach is a principal arranger neurotransmitter in hippocampal cholinergic transmission [30], and the changes of Ach output is positively related to spatial memory performance [31]. From our experimental study, we can postulate that the ethanolic extract promotes Ach concentration thus it is may help the neurogenesis processes on the CA1 and CA3. The sustainability of neurogenesis on CA 1 and CA3 may help or avoid the impairment in cognitive, learning, and memory.

In summary, this study demonstrated daily oral administrations of 100 mg/kg b.w. ethanolic extract *O. sanctum* promotes the increasing of pyramidal cells density on CA1 and CA3 hippocampus. In here also we can rule out the involvement of Ach in the mechanism of neurogenesis thus may stimulate increasing density of neuronal cells in CA1 and CA3.

## Conclusion

Taking together, ethanolic extract *O. sanctum* increase the density of pyramidal cells on CA1 and CA3 hippocampus through upregulated Ach signaling.

## Authors’ Contributions

DLK and HW designed the experiments and study; DLK, HW, and AH performed the experiments; DLK and HW interpreted the data and wrote the manuscript. All authors read and approved the final manuscript.
